# Thirteenth Annual Meeting of the American Medical Association

**Published:** 1860-07

**Authors:** 


### THIRTEENTH ANNUAL MEETING OF THE AMERICAN MEDICAL ASSOCIATION.

### FIRST DAY.

The convention met in the Chapel of Yale College, and at 11 o'clock was called to order—President, Dr. Henry Miller, of Kentucky, in the chair.

Prof. Fisher, of Yale College, opened the convention with prayer.

Dr. Chas. Hooker moved the Committee of Reception report. Dr. Knight, as chairman of the committee, made a most felicitous salutatory to the Convention, and was received with much applause. He spoke ably, and showed the lofty character of such a Convention. His remarks on the progress of medicine, and especially of the art of surgery, were received with marked attention. He reviewed with his remarkable liveliness and interesting manner the wonderful improvements, such as the ligature of the great arteries, the introduction of anaesthetic agents in the greater operations, thus relieving that stinging pain and great anguish to which patients were formerly subjected. He closed by welcoming the profession to the hospitalities of the city, and welcoming them all to open homes and hearts.

Dr. Chas. Hooker then followed in behalf of the Committee of Arrangements. He spoke as follows:

Mr. President, and Gentlemen of the Am. Med. Association:—It is with unwonted gratification that the Committee of

Arrangements welcome you to the City of New Haven. And we only bespeak the common feeling of our fellow citizens in saying that we are delighted—nay, proud—to receive you as our guests. We feel that any city is highly honored to become the chosen place of meeting of the American Medical Association—a select delegated national Congress, representative of forty thousand members of a learned and humane profession. As a city, we appreciate this honor, and should be ungrateful did we not receive you with a generous and cordial welcome. You meet, gentlemen, for a great and noble object—for the promotion of a science vitally linked with the interests of humanity. Your meetings have a most happy influence in strengthening those ties by which the great Fraternity of Medicine are bound in social compact. Another salutary incidental benefit of your meetings, results from their affording an annual period for relaxation and social enjoyment.

Too many physicians prematurely break down in their career of usefulness, in consequence of unremitted and arduous application to their professional duties; and many of you now present, whose exhausted physical and mental energies need recruiting, could hardly have been drawn away from your routine of toil and care, but for your sense of bounden duty to aid in the great object of this Association. We congratulate you, therefore, bretheren, on this annual recurrence of our National Medical Jubilee. In behalf of the faculty of Yale, we welcome you to the halls of this ancient seat of learning, in which you are invited to hold your sessions; and in behalf of the citizens generally of New Haven, we tender you the hospitalities of our city.

We hope that to all of you this meeting will be a season of pleasant social intercourse long to be remembered for the many friendships here formed; and we trust that the harmony and wisdom of your counsels will efficiently promote the great benevolent objects of our organization."

The President ordered as the next business, the calling of the roll. This occupied some half an hour, which the galleries and the Convention to some extent used as a recess.

The whole number of Delegates who answered to their names was between 275 and 300. When all are present the whole number of Delegates will probably exceed the last figure. Twenty-seven States and the District of Columbia are represented in the Convention. There are also a few from the United States Navy.

Dr. Charles Hooker here gave notice of the five divisions and their respective rooms as follows:

1st, Anatomy and Physiology, President's Lecture Room.

2d, Surgery, Geological Cabinet.

3d, Practical Medicine and Obstetrics, Geological Cabinet.

4th, Chemistry and Materia Medica, Chemical Labratory.

5th, Meteorology, Chemical Labratory.

Dr. James Hadley, of Mass., moved that if any Surgeons or Physicians from the Navy be present, they be invited to take seats on the floor of the Convention; carried.

Dr. Jno. Bronson asked if seats had been reserved for the ladies attending the Convention with their husbands; also, for ladies in general.

Dr. Chas. Hooker stated that the galleries were open to the ladies, and the gallantry of the gentlemen would prompt them to make room at all times. The President stated that the Committee on parliamentary rules were ready to report—ordered.

Report was read.

Dr. Brodie moved, before the resolutions be acted on, they be printed.

Dr. Cox moved an amendment, that 500 copies be printed. Amended again by the motion that 1000 copies be printed.

Here an exciting discussion took place in regard to the necessity of having them printed, merely for acting upon, and after an indiscriminate debate, urging the prevalence of pet motions and amendments, a motion to lay the whole affair on the table, prevailed, by a small majority.

A motion for a recess of ten minutes was then carried, the object being to give each State an opportunity to choose its member of the Nominating Committee.

At a quarter before one o'clock, the Convention re-assembled, when the following Committee was declared.

District of Columbia—Dr. Boyle.
Maryland—C. C. Cox.
Kentucky—R. J. Breckenridge.
North Carolina—James H. Dixon.
Tennessee—I. S. White.
Delaware—Lewis P. Bush.
Louisiana—Austin Flint, Jr.
Minnesota—Daniel W. Hand.
Georgia—N. W. Brown.
Massachusetts—D. Humphreys Storer.
Maine—Amos Nourse.
Indiana—Daniel Meeker.
New Jersey—J. S. English.
Rhode Island—James H. Eldridge.
New Hampshire—George H. Hubbard.
Illinois—A. L. McArthur.
Mississippi—U. G. Williams.
Michigan—C. L. Ford.
Pennsylvania—Wilson Jewell.
Iowa—D. L. McGugin.
Ohio—Robert Thompson.
Missouri—M. A. Pallen.
Vermont—Charles L. Allen.
Virginia—James H. Connag.
Connecticut—L. N. Beardsley.
South Carolina—H. R. Frost.
New York—H. D. Bulkley.

A motion was made and carried, inviting the Legislature to be present at the opening of the Convention in the afternoon, to listen to the address of the President, as it would have some reference to medico-legal topics.

AFTERNOON SESSION.

At 3 o'clock, the Convention came together, and notwithstanding the unpleasant weather, the galleries were well filled, including quite a number of ladies.

Convention called to order.

Gov. Buckingham and Lt. Gov. Catlin appeared on the stage, and were introduced to the Convention amid applause.

The Secretary, Dr. Bemis, of Kentucky, then gave the names of the Committee on Credentials.

When the House had become still, President Henry Miller was introduced, and delivered his Valedictory Address. Quite a number of the members of the Legislature were present. Most of it was a bold exposition of personal opinions regarding the moderate and limited standard of medical education. We could see, as he advanced his views regarding preliminary instruction and the duties of Medical Colleges in raising their standards of requirements, that he had the cordial support of the Convention, by the earnest attention and frequent applause attending his suggestions.

The Nominating Committee here reported the names of officers for the Convention, as follows:

President—Eli Ives, Conn.

Vice Presidents—Wilson Jewell, Pa.; A. B. Palmer, Mich.; Joseph P. Logan, Ga.; J. N. McDowell, Mo.

Secretaries not reported.

Treasurer—Casper Wistar, Pa.

The various Committees were then appointed to wait upon the different grades of officers to the stage, as follows:

For escort of President: Jonathan Knight and Dixi Crosby.

For Vice-Presidents: W. C. Snead, of Ky.; Wm. Brodie, of Mich.; Edward Warren, of Md.; R. C. Foster, of Tenn.; H. I. Bowditch, of Mass.: Lewis Sayre, of N. Y.: Jno. L. Atlee, of Pa.; Austin Flint, of La.

Several invitations to visit prominent public places and factories of the city were read, and times set apart for such visits. At 5 o'clock this (Wednesday) afternoon, the Convention, will visit Messrs. G. & D. Cook & Co.'s carriage factory.

Motions were made of acceptance of the invitations, &c.

Motions made to suspend business and receive the officers just elected. They were received with great and prolonged applause.

President Dr. Ives made a very short address, of which the following is nearly a verbatim report:

"All he had, all he was, he owed to his profession. He loved it. He had two sons in the profession, also a grandson; and he, like a very distinguished physician of the present century, could say he would visit the sick as long as he could go, and when he was unable, he would be carried to the bedside."

He was followed by First Vice President, Dr. Wilson Jewell, who will preside over the deliberations of the Convention.

Dr. Davis, of Illinois, offered a series of resolutions to the specific business of morning and afternoon sessions, as follows:

*Resolved*, That the general meetings of the association after this day, shall be restricted to the morning sessions, aud the afternoon sessions, commencing at 3 o'clock, shall be devoted to the hearing of papers and discussions in the several sections.

*Resolved*, That each section shall choose its own officers, and make its own rules of order.

There were other resolutions of this same series which are not reported, because not finally acted upon. The third one relative to the referring of public essays, addresses, &c. to their respective sections, caused a long and exciting debate, in which Drs. Watson, Reese, Miller and Palmer, took a prominent part. After the discussion had run on over an hour, without seeming to come to any mutual understanding in the matter, a motion to table this resolution was almost unanimously carried, with the proviso that Dr. Davis should have an opportunity to revise it, and at his own time to bring it again before the Convention.

Dr. Little, of California was then announced, and although not a regular delegate, was invited to a seat on the floor of the Convention, there being no delegate from California.

A committee on Voluntary Communications was then appointed, viz: Drs. E. D. Foree, of Kentucky; T. W. Blatchford, of New York; N. S. Davis, of Illinois; R. LaRoche, of Pennsylvania; T. F. Rochester, of New York.

At his own request, Dr. LaRoche was excused from serving on this committee.

Dr. Reuchenberger, of Pennsylvania, was appointed in his stead.

The report of the Treasurer was then called for, read and adopted, then referred to Committee on Publication.

The Committee on Publications then reported. Report accepted.

Committee on Prize Essays was called on to report, but failed to do so through absence.

Adjourned.

SECOND DAY.—WEDNESDAY.

The Convention was called to order by the First Vice President, Dr. Wilson Jewell, of Penn.

The minutes of the convention of yesterday were read and approved.

The President announced that the subscription list for the Sydenham Society was on the Secretary's table.

An opportunity was now given for Delegates to name physicians from States not represented, also from the Army and Navy, as members by invitation.

Dr. Gardiner moved that the rules of order be suspended for Dr. Logan, of Ga., to tender his resignation as Vice President. Resignation accepted.

Committee on Education reported—Dr. Reese, Chairman.

This was far the most lengthy and deeply studied report yet made, being a most able exposition of the necessities of our Medical Colleges. We hope to give this in full hereafter. He particularly dwelt on the necessities in preliminary education —Practical Anatomy, Pathology, and Clinical Medicine. He ably supported his argument in favor of lengthened terms of study, with a less number of lectures per day—four being the maximum.

Dr. Bodie moved that the Report and Resolutions connected with it be received and referred to the Committee on Publication; received.

On motion, the House went into Committee of the Whole on the Resolutions—H. F. Askew, of Del., in the chair.

Dr. McDowell of Mo., spoke against the First Resolution, and immediately the galleries were densely crowded, and every effort made to get a sight of the eccentric Missourian. Almost every sentence drew forth roars of laughter. He was loudly cheered, and often interrupted by the repetition of applause.

Dr. Henry Miller, of Ky., replied.

Dr. Palmer, of Michigan, continued the discussion.

Motion made that the whole subject be laid on the table; lost.

Motion made that the Committee rise, report progress, and ask leave to sit again. Carried.

The Committee on Nominations reported that the Convention will meet at Chicago on the 1st Tuesday in June, 1861. Amendment offered that it be changed to the 1st Tuesday in May.

Dr. Davis of Illinois, spoke for the Illinois delegation, urging June as the proper month—furthermore, he welcomed the Convention to the hospitalities of the citizens of Chicago.

Motion made to change the time to the 2d Tuesday of June; unconstitutional. Original report adopted.

The Committee on Nominations here concluded their report as follows:

In place of 3d Vice President, Dr. Logan, of Ga., resigned, Dr. R. D. Arnold, of Georgia.

Secretaries—S. G. Hubbard, Ct.; H. A. Johnson, Illinois.

Committee of Arrangements—N. S. Davis, J. W. Freer, DeLaskie Miller, E. Andrews, H. W. Jones, Thos. Bevan, J. Bloodgood, all of Illinois.

Prize Essays—Daniel Brainard, Ill.; D. L. McGugin, Iowa; M. L. Linton, Mo.; John Evans, Ill.; A. L. McArthur, Ill.

Committee on Publication—F. G. Smith, Penn.; Casper Wistar, Penn.; S. G. Hubbard, Conn.; R. J. Breckenridge, Ky.; Ed. Hartshone, Penn.; H. F. Askew, Del.

The report was adopted.

Dr. Davis, of Ill., called for a suspension of the rules, that he might re-introduce his resolution laid upon the table yesterday. Carried. The resolution having been revised, was reported and carried as follows, viz:

*Resolved*, That all Essays, volunteer communications, and reports, except those from the officers of the Association and the Committees on Medical Education, Medical Literature, and Prize Essays, shall be first presented to the association and referred to the appropriate sections, in which they shall be examined and discussed, after which they shall be returned to the Secretary of the Association with a recommendation accompanying such as are deemed worthy of Publication ; and the Secretary shall pass all such as are thus recommended directly to the Committee of Publication, and such as are not recommended by the Section as worthy of Publication, shall be retained by the Secretary or returned to their authors as the latter may indicate.

Report of Committee on Prize Essays, was called for.—Prof. Worthington Hooker, of Conn., Chairman.—Three Essays had been handed in—two of which had considerable merit, and showed much research. But neither of them were of such a character as would justify the award of a prize. Accepted.

Moved, a suspension of the rules, to give Dr. Wilbur, of N. Y., an opportunity to report the protest of Ignatius Langer, of Iowa, against the action of the Committee of Arrangements in not accepting his credentials as a delegate. The President stated he held in his hand a letter stating that Dr. Langer had been expelled from the Scott County Medical Society of Iowa, and therefore the rules of the Society would not permit his acceptance as a delegate here.

Motion to suspend lost, almost unanimously.

Reports of Special Committees were then called for, and disposed of as follows:

Morbus Coxarius and Surgical Pathology of Articular Inflammation—Lewis A. Sayre, N. Y. ; referred to the section on Surgery.

Surgical Treatment of Strictures of the Urethra—James Bryan, Penn., reported progress and asked for longer time ; referred to its proper section.

Drainage and Sewerage of large cities, their influence on Public Health—A. J. Semmes, Cornelius Boyle, G. M. Dove, D. C. ; reported progress and asked for longer time.

Puerperal Tetanus : its Statistics, Pathology and Treatment —D. L. McGugin, Iowa ; report the same as above. Requested to report progress in the section on obstetrics &c.

Hospital Epidemics—R. K. Smith, Penn. ; laid over.

Puerperal Fever—S. N. Green, Indiana ; laid over.

Anaemia and Chlorosis—H. P. Ayers, Indiana ; reported progress and asked to continue the Committee to report next year.

Veratrum Viride—J. B. McCaw, Va; laid over.

Alcohol: Its Therapeutical Effects—J. W. Dunbar, Md.; asked for a change in the title, making it read, "Alcohol in its relations to man"—granted. Report next year.

Meteorology—J. G. Westmoreland, Georgia; laid over.

Milk Sickness—Robt. Thompson, Ohio; partial report made —accepted and referred to section of Practical Medicine.

Manifestations of Disease of Nervous Centres—C. B. Chapman, Wis.; laid over.

Microscopic Observations on Cancer Cells—Geo. N. Norris, Ala. Chairman asked to resign; Committee discharged.

Philosophy of Practical Medicine—James Graham, Ohio; laid over.

On some of the Peculiarities of the North Pacific and their relations to Climate—William H. Doughty, Georgia. Referred to section on Meteorology, &c.

On the Microscope—John C. Dalton, Jr., N. Y., David Hutchinson, Ind., A. R. Stout, Cal., Calvin Ellis, Mass., Christopher Johnston, Md.; report next year.

Diseases and Mortality of Boarding Schools—C. P. Mattingly, Ky.; Dixi Crosby, N. H.—reported progress; referred to its proper section.

On Various Surgical Operations for Relief of Defective Vision—M. A. Pallen, Mo., T. J. Cogley, Ind., W. Hunt, Penn.; laid over.

On the Blood Corpuscle—W. Sager, Michigan; granted additional time.

American Medical Necrology—C. C. Cox, Md. Report was ordered to be read before the Convention, Thursday.

Effects of the Virus of the Rattlesnake when Introduced in the System of Mammalia—A. S. Payne, Va.; no report and Committee discharged.

Constitutional Origin of Local Diseases, and the Local Origin of Constitutional Diseases—W. H. McKee, N. C., C. F. Haywood, N. C., C. F. Haywood, N. Y.; laid over.

Subcutaneous Injections as Remedials—I. Langer, Iowa; not allowed to report, not being an accepted delegate.

Quarantine—D. D. Clark, Pa., E. M. Snow, R. I., W. Jewell, Pa., E. D. Fenner, La., I. W. Houck, Md.; asked to be continued. Agreed to.

Medical Ethics—B. F. Schenk, Pa., chairman. Resigned, and asked that Dr. Paul F. Eve, of Tenn., be substituted; agreed to. Report next year.

Tracheotomy in Membranous Croup—A. V. Dougherty, N. J. Partial report; this was accepted, and referred to the Surgical Section. Further time allowed to make out the report.

Effect of Perineal Operations for Urinary Calculi upon Procreation in the male; J. S. White, Tenn. Letter from Dr. White read; laid over.

Mercurial Fumigation in Syphilis—D. W. Yandell, Ky.; laid over.

Cause and Increase of Crime—W. C. Snead, Ky.; asked to be continued. Agreed to.

Education of Imbecile and Idiotic Children—H. P. Ayres, Ind. Report offered; referred to its proper Section.

Pons Varolii—Partial report. The Committee wished to be continued; agreed to. Referred to Section on Anatomy.

One o'clock, the hour of adjournment, having arrived, a motion to continue five minutes longer prevailed. A little general business was then transacted, and the Convention adjourned.

AFTERNOON SESSION.

In accordance with the plan adopted at the Annual Meeting of the Association in Louisville, and further provided for by the resolutions introduced by Dr. N. S. Davis during the present session, the members of the Association assembled at 3 o'clock, P. M., in the rooms provided for the several Sections.

As might have been anticipated, the Sections on Surgery, Practical Medicine, and Obstetrics, and Meteorology and Epidemics, attracted the chief attention. That on Surgery was most numerously attended. Dr. L. A. Sayre, of New York, read a very important and elaborate paper on the Pathology and Treatment of Morbus Coxarius or Hip-Disease. Among the points of chief practical importance presented in the paper were the results of examinations showing that many of the cases of this disease are not connected with *tubercular deposits*, as had been too confidently asserted by some; the advocacy of early incisions into the joint for the discharge of such serous and purulent fluids as generally accumulate in the earlier stages of the disease; the exsection of the head of the bone whenever it was found denuded and carious; and throughout the whole treatment, the application of an apparatus for maintaining sufficient extension to prevent all pressure of the head of the femur against the upper part of the acetabulum, and yet allow the patient to take some exercise. All the important practical deductions of the writer were sustained and illustrated by cases reported in detail. The reading of this paper, and the exhibition of the apparatus accompanying it, was followed by a very interesting discussion, which was participated in by a large number of the more eminent practical surgeons in our country, among whom we noticed Drs. Willard Parker, J. P. Batchelder, J. R. Wood, Alden March, and F. H. Hamilton, of New York; J. McDowell of Missouri; J. Knight, of Conn., and others.

The paper was universally regarded as possessing great practical value, and was recommended for publication in the Transactions of the Association.

About fifty members of the Association assembled in the Section on Practical Medicine and Obstetrics, and organized by the election of Dr. Nourse, of Maine, Chairman, and Dr. A. K. Gardner, of New-York, Secretary. A voluntary communication, entitled, "Treatment of Phthisis by Chlorate of Potassa, with observations on Oxygen and Ozone as Therapeutical Agents," was read by the author, Dr. Fountain, of Davenport, Iowa.

The author assumed that *tubercle* was the result of an imperfect oxydation and metamorphosis of the nitrogenous structures of the body, and therefore, whatever would increase the oxygen of the blood would not only act as a prophylactic, but would also furnish the conditions most likely to effect the disintegration and ultimate excretion of the tuberculous matter already formed. The well-known efficacy of the Chlorate of Potassa in increasing the arterial hue of the blood, and increasing its capacity for oxygen, suggested its use as a remedy for tuberculosis. The author reported only three cases treated with the Chlorate, but in each of these its effects were represented as remarkably beneficial. He gave it to the extent of half an ounce, dissolved in water every twenty-four hours. The paper also contained a careful review of the facts hitherto recorded in relation to *Ozone*, and its connection with oxygen, from which the author was led to the conclusion that the former was simply oxygen in a *nascent* state. The paper was a highly interesting and suggestive one, and led to a discussion in which many members participated. During the discussion, the fact was elicited that a large proportion of those present had used Chlorate of Potassa, and found it highly valuable in Scarlatina, Diphtheria, Typhus Fever, and various diseases supposed to be accompanied by a depressed condition of the vital properties, but very few had administered it in tuberculosis. If we remember correctly, Dr. Wooster, of New York, stated that he had used it in some cases of phthisis with decided benefit; and Dr. N. S. Davis, of Chicago, stated that he had in several instances prescribed it in the advanced stage of phthisis, when from the feebleness of the patient, and the diminished capacity of the lungs, he becomes much oppressed and the blood venous, as indicated by blueness of the lips and nails; and almost always with much temporary relief to the patients. But he had never used it as a curative agent in the early stage of tuberculosis, nor in quantities so large as recommended in the paper. In view of the importance of the subject, and the small number of cases reported in the paper of Dr. Fountain, the following resolution was proposed and adopted:

*Resolved*, That the Section has listened to the paper of Dr. Fountain with deep interest, and suggest that it be referred back to the author, with the request that he continue his investigations, and report to the next annual meeting of the Association.

Dr. N. S. Davis, of Chicago, then read a full abstract of a paper on the Effects of Alcoholic Stimulants on the Developments of Tubercular Diseases. The paper was founded on the history of 210 cases of phthisis collected by the author during the last five years, and the conclusions to which he arrived are as follows, viz:

1st. That the development of Tubercular diseases is facilitated by all those agents and influences, whether climatic or hygienic, which directly or indirectly impair or retard the metamorphosis of the organized structures and the efficiency of the excretory functions.

2d. That both observations and carefully devised experiments show that the presence of Alcohol in the human system, notwithstanding its temporary exhileration of the nervous system, positively retards both metamorphosis and elimination.

3d. That, neither the action of alcohol on the functions of the human body, nor the actual results of experience, furnish any evidence that alcoholic beverages are capable of either preventing or retarding the development of tubercular phthisis.

The reading of the abstract was followed by some remarks by Drs. Austin Flint, of New York, Bowditch, of Boston, and others; when on motion the paper was recommended to the Association as worthy of publication.

The Section on Meteorology, Epidemics, etc., was organized by the appointment of Dr. C. A. Lee, Chairman.

A very elaborate and valuable paper was presented to this Section, on the Topography and Epidemics of New York State, by Dr. Joseph M. Smith. It was recommended by the Section for publication in the Transactions of the Association.

A report was also presented on the Topography and Climate of some portion of California, which, after some discussion, was recommended to be returned to the author, chiefly from a deficiency of statistical and tabulated facts.

On motion, the Section adjourned to 3 o'clock the following day.

THIRD DAY.—THURSDAY.

The Convention was called to order at 9 o'clock—the President, Eli Ives, M. D., of Conn., in the chair.

The minutes of the previous day's proceedings were read by the first Secretary, Dr. S. G. Hubbard, of Conn.

A motion to accept the Secretary's report without reading, was lost. The minutes were accepted.

A list of recent registrations was then read. There are now registered between 550 and 600 delegates.

Dr. Chas. Hooker spoke of the number registered, and that for some reason unknown, many delegates did not register themselves at all, as well as many permanent members—and that many registered themselves without signing the Constitution.

Dr. Arnold, of Ga, to facilitate business, offered a rule that no member be allowed to speak or read addresses of more than ten minutes in length; carried.

Dr. Shattuck moved a suspension of the rules for the purpose of introducing two resolutions; carried.

Dr. Bowditch reported resolutions on the Hunter Memorial to be erected in Westminster Abbey; accepted.

Report of the committee appointed to confer with the American Medical Teachers' Convention. Report received and adopted section by section. The resolutions were discussed at some length by Drs. Flint, of New York, Shattuck, of Mass., McDowell, of Mo., Atlee, of Penn., Brodie, of Mich., Palmer, of Mich., Nourse, of Me.

Dr. Bennett, of Danbury, Ct., moved that the whole be laid on the table; lost.

Dr. Johnson, of Mo., continued, followed by Worthington Hooker, of New Haven, N. S. Davis, of Ill., Reese, of New York, Humphreys Storer, of Boston, and Mussey, of Cincinnati.

All the amendments to the fourth resolution were withdrawn, and the previous question ordered.

Dr. Childs, of Mass., took the stand for an explanation. Dr. Storer, amid great confusion, rose to a point of order. Ruled out. Great excitement, a dozen attempting to get the floor, amid shouts of "go on," "hear him," &c.

Dr. Shattuck spoke regarding the 4th resolution.

Dr. W. Hooker made an explanation regarding the Yale Medical College, and its connection with the State Medical Society,

Dr. Watson, of N. Y., spoke against the 9th resolution. It was laid on the table. It virtually cut off the New York Academy of Medicine from this Society.

Dr. Atlee spoke of it as altogether unconstitutional, and the law was read—" Each local society shall have the privilege of sending to the Association one delegate for every ten of its regular resident members, and one for every additional fraction of more than one half this number. The Faculty of every regularly constituted Medical College or chartered school of medicine shall have the privilege of sending two delegates.

" The professional staff of every chartered or municipal Hospital, containing an hundred inmates or more, shall have the privilege of sending two delegates; and every other permanently organized Medical Institution, of good standing, shall have the privilege of sending one delegate."

The 9th resolution was laid on the table.

The whole report was adopted, and referred to committee of Publication.

Committee on Nominations reported—

Committee on Medical Literature: Frank H. Hamilton, New York; Edward Warren, Md.; Chas. A. Lee, New York; I. W. C. Ely, R. I.; E. H. Clark, Mass.

Committee on Medical Education:—L. S. Ayres, Va.; C. C. Cox, Md.; I. C. Bradbury, Maine; L. H. Steiner, Md.; M. A. Pallen, Missouri.

Surgical Treatment of Stricture of the Urethea—James Bryan, Pennsylvania.

Drainage and Sewerage of large cities:—A. J. Semmes, La.; C. Boyle, Ga.; W. C. Dove, District of Columbia.

Puerperal Tetanus statistics, Pathology and treatment—D. L. McGugin, Iowa.

Anœmia and Chlorosis—H. P. Ayres, Ind.

Alcohol and its relations to man—J. W. Dunbar, Maryland.

Milk Sickness—Robert Thompson, Ohio; S. M. Beemis, Ky.

On the effect of Perineal Operations for Urinary Calculi upon pro-creation in the male—I. S. White, Tenn.; J. B. Mc Caw, Va.; R. C. Foster, Tenn.

Mercurial Fumigations in Syphilis—L. W. Yandell. Ky.

Cause and Increase of Crime—W. C. Snead.

[We are obliged to omit the remainder of the Committees. We append the Resolutions:]

*Resolved*, That it be recommended to the different States to collect subscriptions of not more than one dollar each from every regularly educated physician. All monies so collected to be forwarded by the chairman of the committee here, by appointment, to the Treasurer of the Hunter Memorial in London.

*Resolved*, That Drs. Henry Bowditch, of Mass., Amos Nourse, of Me., G. B. Twitchel, of N. H., C. Clark, of Vt., G. L. Collins, of R. I., Chas. Hooker, of Conn., and many others be a Committee to collect subscriptions.

Resolutions adopted as a whole.

Moved that a copy of these Resolutions be sent to all regular Medical Colleges of the country—carried.

Resolution made and accepted that a seal of this Society be given to every Medical School in good standing, to be withdrawn upon evidence of misconduct.

Motion made to meet in mass this afternoon at 4 o'clock; carried.

Amidst great confusion and the putting of motions and amendments a motion to adjourn was lost. Motion made and carried that the Sections meet at 2½ P. M. Adjourned.

AFTERNOON SESSION.

[*Note.*—Jno. L. Atlee, of Pa., was substituted on the Nominating Committee in place of Dr. Wilson Jewell, who was elected one of the Vice Presidents. The above in our yesterday's report was omitted by accident.—Ed.]

The Association was called to order by the First Vice President.

The President requested the Committee on the Hunter Memorial to retire for private business.

Moved that Dr. White be permitted to continue his report.

Report of Committee on Medical Typography and Epidemic Diseases referred to the Committee on Publication.

Committee on Hospital Epidemics discharged.

Committee on Puerperal Fevers discharged.

Committee on Veratrum Viride discharged.

Report on Improvements in Surgery referred to the Section on Surgery.

Report on Inebriate Asylums referred to the committee on Publication.

The President called for a report of each of the Sections.

1st. Anatomy and Physiology; referred to the committee of Publication.

2d. Practical Medicine and Obstetrics; report referred to Committee of Publication.

3d. Section on Surgery; report adopted; ordered Published.

4th. Meteorology; report adopted and referred to the committee on Publication.

The President called for a report of the committee on Rules of Order.

The Report was taken up seriatim.

After much discussion, a motion prevailed to lay all the Rules of Order on the table.

Dr. McDowell, of Mo., moved that the Convention go into a Committee of the Whole for the purpose of discussing Dr. Reese's resolutions; withdrawn by request.

Resolutions from the Essex Co. Medical Society of N. J. were offered; adopted.

Moved that a Special Committee be appointed to confer with the different legislatures on this subject.

Motion made and carried that Dr. Cox be continued on the committee of Necrology.

Report of the committee on Tracheotomy was read; adopted. Referred back to committee to continue and report next year.

Dr. Bell, of Brooklyn, introduced a resolution giving the Sections power to refer papers to experts to determine whether they be published; amended to be referred to the committee of Publication.

Whole matter laid on the table.

A communication from the Judicary Committee of the Connecticut Legislature was read, asking that a committee be appointed to report a bill upon the subject of Criminal Abortion, for action at the next session—carried.

The Chair will appoint a Committee in due time.

Moved that the American Medical Teachers Convention be perpetuated in connection with the American Medical Convention, and delegates be appointed to meet from each Medical School, the day before the American Medical Convention, at the same place.

Amended to "meet regularly" instead of being perpetuated —adopted.

Moved that the committee of last year, on this subject, be continued.

Moved by Dr. Atlee that the Hunterian Committee be empowered to fill all vacancies in it—carried.

Resolution offered that a committee be appointed to prepare rules for this organization—laid on the table.

President Day, of Yale College, was invited to a seat on the stage.

Communication from Elmira, N. Y., read. Referred to Surgical Section.

Moved and carried that a vote of thanks be offered to Dr. Beemis for the faithful discharge of his duties as Secretary; amended by substituting "Retiring Officers."

Resolution offered of thanks from this Association to the Faculty of Yale College and to the citizens of New Haven, for their elegant hospitalities and kindness during their stay here—carried unanimously.

The three amendments to the Constitution proposed last year by Dr. Mason and Dr. Lindsby, were then taken up. The first, relating to the appointment of delegates, was rejected. The second, requiring all permanent members to present certificates of good standing, received a large vote, but not the constitutional majority of *three-fourths.*

The third amendment, proposed by Dr. Lindsby, was indefinitely postponed.

Dr. Hooker spoke to the Convention in regard to commutation tickets.

Moved that they go into a Committee of the Whole; carried. Dr. Askew in the chair.

A discussion was called up in regard to the Resolutions of Committee of Education, Dr. Reese, chairman.

Dr. Gardiner moved the Committee rise, report progress, and report the resolutions entire to the Committee of Publication.

Dr. Hamilton, of Brooklyn, offered a resolution for a bill for the establishment of a College of Physicians and Surgeons of American Medical Association. Discussed by Drs. Hamilton, Gardiner, and others. Resolution withdrawn.

Dr. Cox, of Md., spoke at length in favor of the primary resolutions.

Dr. Atlee introduced Dr. Stephens, Ex-President of the Convention. He was invited to a seat on the stage.

Dr. Neringer, of Pa., spoke against the second resolution.

Dr. Hamilton supported his resolution.

Dr. Crane spoke against it.

Moved the Committee rise and refer the resolutions to the Convention; carried.

Vice-President Askew here presented the report of the Committee of the Whole to the Convention. Accepted. Referred to Committee of Publication.

Dr. Dixi Crosby addressed the Convention as to its general action.

Motion made that the Convention adjourn *sine die*. Carried.

DOINGS IN THE SECTIONS.—THIRD DAY.

At 2½ P. M., of Thursday, the members of the Association again assembled in the several Sections. As on the previous day, the Section on Surgery was most numerously attended, and several papers of importance were considered and disposed of.

The Section on Practical Medicine and Obstetrics was well attended, and as the whole time of the previous session was occupied with papers in relation to Practical Medicine, those relating to Obstetrics were given the precedence. An interesting and profitable discussion took place concerning the treatment of retro-version of the Uterus, which was participated in by many of the members, most of whom spoke favorably of the use of the elastic air-bag as an aid in effecting replacement. Dr. McGugin, of Iowa, made a partial report on the subject of

Puerperal Tetanus. He stated that he had collected reports of twelve or more cases, in addition to those reported by Prof. Simpson ; that a majority of the cases had occurred after *abortions*, accompanied by so much hæmorrhage as to require the use of the tampon ; and that three of the cases had occurred under his own observation.

Dr. Storer, of Boston, expressed some surprise at the number of cases collected by Dr. McGugin, as he had never met with but one case, which occurred after delivery at the full period, and during the retention of an adherent placenta.

Dr. A. K. Gardner, of New York, also thought the disease of very rare occurrence, as he had never met with a case in that city.

A gentleman, whose name we did not get, related a case occurring in his practice, which took place after confinement at the full period, and seemed to arise from imprudent exposure of the feet to a current of cool air, coupled with mental depression from domestic trouble.

Dr. Palmer, of Mich., and several others, made some observations on the subject. As it appeared that a large majority of the cases occurred, either when there was partial or complete retention of the placenta, or after the use of the tampon to suppress hæmorrhage, Dr. Davis, of Ill., inquired whether it was not probable that the disease originated from a poison generated by the decomposition of retained clots, parts of placenta, etc., in the uterus. The subject was discussed with much interest until the hour of adjournment.

In the Section on Meteorology and Epidemics, Dr. Thompson, of Ohio, made a partial report on Milk Sickness, which led to a very interesting interchange of views in relation to that disease, and to the prevalent " cattle disease," or pleuropneumonia at present so fatal in Massachusetts.

For further comments on the general aspect and results of the recent meeting of the Association, see Editorial in the present number of the *Examiner*.

